# Flexible and Twistable ZnMn_2_O_4_‑Electrodeposited Yarn Supercapacitors for Wearable Electronics

**DOI:** 10.1021/acsami.5c06545

**Published:** 2025-06-25

**Authors:** Shalu Rani, Gaurav Khandelwal, Abhinav Tandon, Sanjay Kumar, Akshaya Kumar Aliyana, Suresh C. Pillai, George K. Stylios, Nikolaj Gadegaard, Daniel M. Mulvihill

**Affiliations:** † Department of Electronics Engineering, 28692 Indian Institute of Technology (Indian School of Mines), Dhanbad, Jharkhand 826004, India; ‡ James Watt School of Engineering, 150841 University of Glasgow, Glasgow G12 8QQ, U.K.; § Department of Mechanical Engineering, College of Design and Engineering, 37580 National University of Singapore, Singapore 117575, Singapore; ∥ Research Institute for Flexible Materials, 3120 Heriot-Watt University (Scottish Borders Campus), Galashiels TD1 3HF, U.K.; ⊥ Nanotechnology and Bio-Engineering Research Group, Department of Environmental Science, 8811 Atlantic Technological University, Sligo F91 YW50, Ireland

**Keywords:** flexible supercapacitor, ZnMn_2_O_4_, electrodeposition, bendable and twistable electrodes, wearable electronics

## Abstract

The growing demand for wearable electronics has driven interest in flexible fiber-based supercapacitors (F-SCs) as power sources, offering tunable sizes, adaptable shapes, and versatile design possibilities. This study presents the fabrication of a highly flexible and twistable fiber-shaped yarn supercapacitor (F-SC) via direct electrodeposition of ternary metal-oxide nanostructures (ZnMn_2_O_4_) onto flexible and conductive carbon yarn substrates. The uniform growth of ZnMn_2_O_4_ nanostructures on the carbon yarn not only enhances the capacitive performance of the fabricated devices but also significantly enhances the mechanical integrity of the electrodes, ensuring excellent bending and electrochemical stability for the F-SC device. The device exhibits a high areal capacitance of 87.6 mF/cm^2^ at a scan rate of 10 mV/s and 35.4 mF/cm^2^ at a current density of 0.1 mA/cm^2^. Furthermore, it retains 92% of its capacitance after 10,000 charge–discharge cycles, achieving energy and power densities of 11 μWh/cm^2^ and 385 μW/cm^2^, and maintaining consistent performance under varying bending and twisting conditions. This work offers a simple, cost-effective, and efficient strategy for developing flexible and twistable fiber electrodes using a straightforward electrodeposition process. The fabricated electrodes hold great potential in developing flexible energy storage technologies and enabling seamless integration into next-generation portable and wearable electronics.

## Graphical abstract

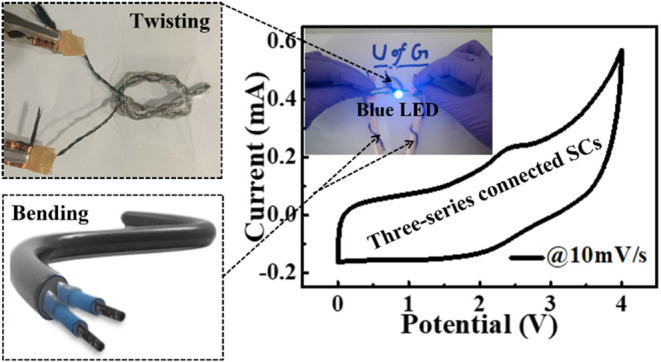

## 1 Introduction

Significant research efforts over the past few decades into flexible energy storage technologies have contributed to the rapid evolution of supercapacitors (SCs) with fast charge–discharge capabilities, superior power densities, and excellent cyclic stability for portable and wearable applications.^1^ Among various SC configurations, flexible and wearable electrodes have attracted considerable interest due to their ability to integrate seamlessly into everyday applications and the possibility of being integrated with commonly used textiles.^2,3^ However, most flexible SCs employ planar or sandwiched structures, limiting their flexibility to two dimensions. Compared to two-dimensional planar energy storage devices, which utilize planar substrates (poly­(ethylene terephthalate)) (PET), polyimide (PI),^4^ carbon paper,^4,5^ carbon cloth,^5^ titanium foil,^6^ nickel foam,^6^ etc., fiber-shaped structures offer distinct advantages, including light weight, compact volume, easy integration, and better portability. These advantages facilitate their integration into complex smart electronic systems such as smart garments and electronic textiles. Furthermore, with the rising demand for wearable energy storage and portable electronics, fiber-shaped SCs have gained considerable attention because of their knittability, flexibility, and durability. These characteristics enable their incorporation into a wide range of everyday fabrics, including smart clothing, gloves, wristbands, and even next-generation interactive garments and accessories.^7^ Despite these advantages, most previously reported flexible fiber-shaped yarn supercapacitors (F-SCs) have been fabricated through complex procedures and often lack mechanical stability and flexibility, both of which hinder their broader application.^8–10^ Consequently, a significant challenge remains in developing approaches that simultaneously enhance the performance and mechanical stability of SC devices while ensuring simplicity, cost-effectiveness, and scalability.

Given futuristic demand, ongoing research is extensively exploring transition-metal oxides (TMOs) as active materials in flexible F-SCs for smart textiles because of their relatively high electrochemical performance, particularly in terms of capacitance and energy density.^10,11^ Among these, ruthenium oxide (RuO_2_) is an ideal electrode material for flexible SC applications, which offers high capacitance and excellent cyclability.^12^ However, its commercialization is hindered by its high cost, toxic nature, and the tendency to form large agglomerates during electrochemical redox reactions.^2^ As compared to RuO_2_, manganese (Mn)-based TMOs, such as MnO_2_,^13^ Mn_2_O_3_,^14^ and Mn_3_O_4_,^15^ present promising alternatives due to their cost-effectiveness, environmental sustainability, and the presence of multiple oxidation states (ranging from +2 to +7), which contribute to high specific capacitance in electrochemical energy storage applications. Nevertheless, their practical implementation is hindered by their poor electrical conductivity and volumetric changes during charging–discharging. To address these limitations, recent research has shifted toward ternary mixed transition-metal oxides (MTMOs), which exhibit enhanced electrochemical properties owing to their AB_2_O_4_ composition (where A and B are transition-metal oxides).^16^ These ternary metal oxides have garnered significant interest due to their diverse features in various applications, including photocatalysis,^17^ sensors,^18^ Li-ion batteries (LIBs),^19^ magnetic applications,^20^ etc. Among various mixed manganese oxides, ZnMn_2_O_4_ has demonstrated exceptional energy storage potential, featuring Zn^2+^ ions at tetrahedral sites and Mn^3+^ ions at octahedral sites. As an energy storage material, ZnMn_2_O_4_ offers several advantages like high energy density, low operating potential, nontoxicity, natural abundance, low cost, environmental compatibility, and commercial viability.^21^ Additionally, ZnMn_2_O_4_ is expected to follow a reaction mechanism similar to that of MnO_2_, involving the surface adsorption of electrolyte cations and/or the insertion/deinsertion of cations within its vacant structural sites during redox transitions. Ternary ZnMn_2_O_4_ has been extensively utilized as an electrode material in diverse applications, including LIBs,^22^ SCs,^23,24^ sensors,^25^ etc. To the best of our knowledge, the utilization of ZnMn_2_O_4_ as an electrode material to develop flexible yarn SCs has not been previously reported.

Various synthesis techniques, including solvothermal,^26^ sol–gel,^27^ hydrothermal,^28,29^ electrospinning,^30^ and coprecipitation^31^ processes, have been employed to fabricate ZnMn_2_O_4_ nanomaterials with diverse microstructures in energy storage applications. These methods typically involve multiple stages, including synthesis of nanomaterials, slurry fabrication, including binders and conducting agents, and subsequent electrode fabrication for the development of SC devices. Among these synthesis techniques, electrodeposition is a widely recognized one-step approach for the nucleation and growth of oxide nanostructures over conductive substrates.^32^ The direct electrodeposition of manganese dioxide layers onto electrodes has proven to be an efficient method for producing high-performance electrode materials, particularly for SC applications. Furthermore, compared to naturally occurring and chemically synthesized manganese oxides, electrochemically grown ZnMn_2_O_4_ on conductive substrates exhibiting enhanced electrochemical characteristics suggests its potential for advanced fiber-shaped energy storage devices.

Because of the promising advantages of ternary metal oxides, this study aims to synthesize ZnMn_2_O_4_ nanostructures using a one-step direct electrodeposition technique within a three-electrode system onto conductive and flexible carbon yarns for the development of F-SCs. This method offers several benefits including precise reaction control, reproducibility, rapid processing, and environmental sustainability. Unlike conventional synthesis techniques, which often require multiple processing steps and the use of binders or additives that may reduce the conductivity and electrochemical performance of the electrode material, electrodeposition facilitates direct and uniform growth of active materials on conductive substrates, ensuring strong adhesion and efficient charge transport. The ZnMn_2_O_4_ nanostructures grown on carbon yarn exhibit an interconnected network morphology, which plays a key role in improving the electrochemical performance and mechanical stability of the device. This unique structural arrangement facilitates rapid ion diffusion and efficient electrolyte penetration, enabling the ZnMn_2_O_4_-grown carbon yarn electrodes to achieve a high specific capacitance and excellent cycle life when employed in yarn-based SC devices. Additionally, the uniform and porous interconnected ZnMn_2_O_4_ nanostructures accommodate volumetric expansion during charge–discharge cycles, which is a common limitation in metal-oxide-based electrodes, thus enhancing long-term cycling stability. Furthermore, the fabricated flexible F-SC device utilizing ZnMn_2_O_4_-grown carbon yarn electrodes demonstrates superior adaptability to bending and twisting, making them promising candidates for next-generation wearable electronics systems.

## 2 Experimental Section

### 2.1 Materials

Zinc acetate dihydrate (Zn­(CH_3_CO_2_)_2_·2H_2_O, 98%), manganese acetate dihydrate (Mn­(CH_3_COO)_3_·2H_2_O, 99.9%), poly­(vinyl alcohol) (PVA), phosphoric acid (H_3_PO_4_), acetone (99.9%), and ethanol were procured from Sigma-Aldrich in their original analytical reagent-grade forms and were used without additional purification.

### 2.2 Electrodeposition of ZnMn_2_O_4_ on Carbon Yarn

To fabricate flexible electrodes for application in F-SC devices, ZnMn_2_O_4_ was directly grown on a conductive and flexible carbon yarn substrate by using the electrodeposition technique (Figure 1). Commercially available carbon yarn was utilized as the current collector. It was initially cleaned using deionized (DI) water and acetone to eliminate surface impurities. The cleaned carbon yarns were then dried in an oven to ensure the complete removal of moisture (Figure 1a). The electrolyte for electrodeposition was prepared by mixing a 1:2 molar ratio of 10 wt % of 0.05 M Zn­(CH_3_CO_2_)_2_·2H_2_O and 10 wt % of 0.05 M Mn­(CH_3_COO)_3_·2H_2_O in DI water under constant stirring. The reagents were continuously mixed until a homogeneous solution was obtained. The electrodeposition was performed in a three-electrode setup with the cleaned carbon yarn acting as the working electrode, Ag/AgCl as the reference electrode, and a platinum wire as the counter electrode, all immersed in the prepared electrolyte solution. The deposition process was conducted using cyclic voltammetry (CV) for 25 and 50 cycles at a 50 mV/s scan rate within the potential range between 0 and 1 V, using a Metrohm Autolab electrochemical workstation. Following the electrodeposition process, the ZnMn_2_O_4_-coated carbon yarn electrodes were thoroughly washed with DI water and ethanol to remove the residual reactants and subsequently dried at 60 °C for 6 h, as displayed in Figure 1b,c. The resulting ZnMn_2_O_4_-deposited carbon yarn electrodes (at 50 and 25 cycles named ZMO@carbon yarn and ZMO1@carbon yarn, respectively) were subjected to further characterization and utilized for SC device fabrication.

**Figure 1:**
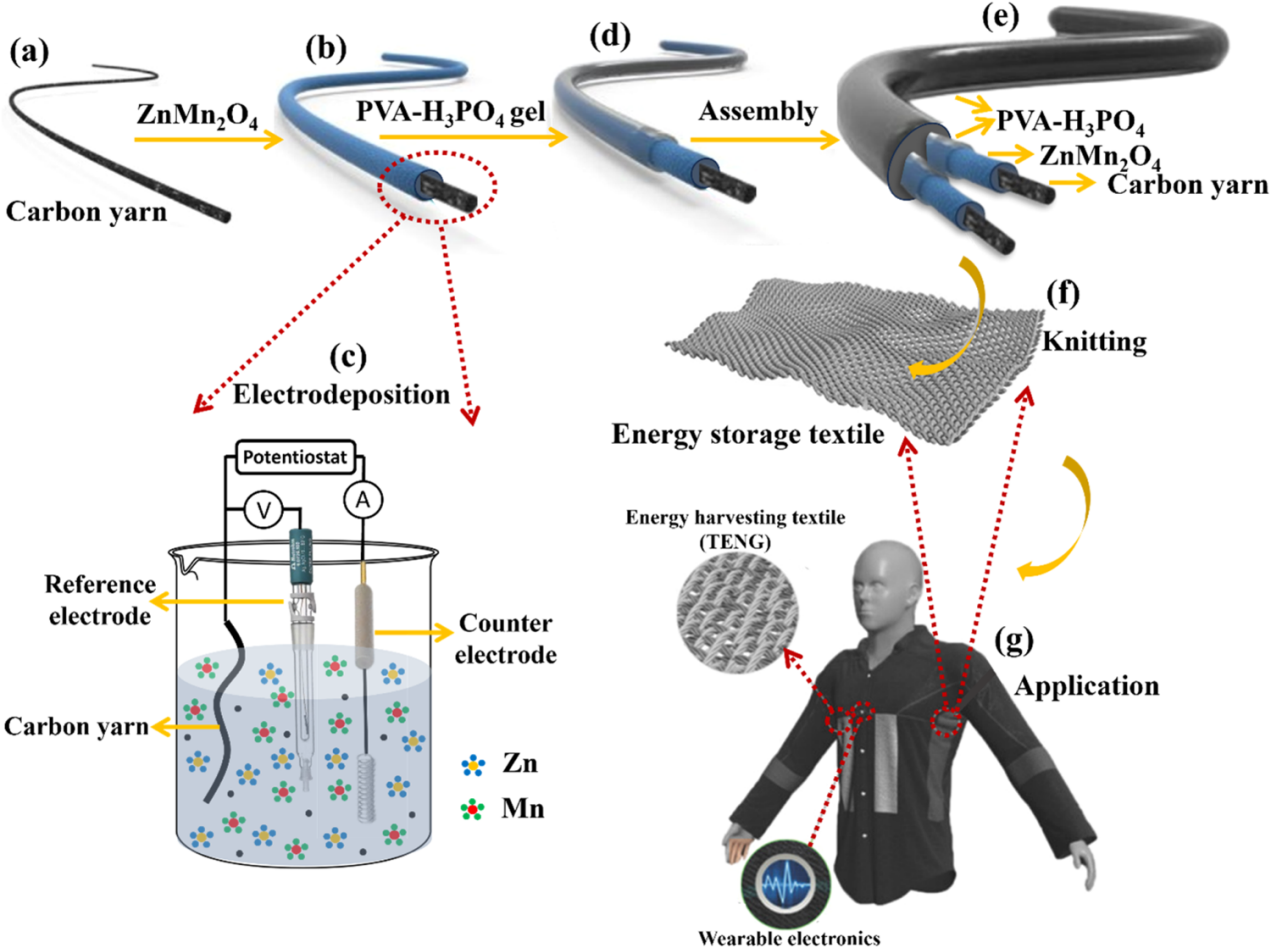
Fabrication process flow for F-SC device: (a) Bare carbon yarn, (b) ZnMn_2_O_4_-grown carbon yarn, (c) electrodeposition process to deposit ZnMn_2_O_4_ over carbon yarn, (d) PVA-H_3_PO_4_ gel electrolyte coated over ZnMn_2_O_4_-grown carbon yarn, (e) assembly of flexible F-SC device with gel electrolyte coating, (f) knitting of fabricated flexible F-SC device, (g) application example of the fabricated device in wearable electronics. Panel (g) is reproduced with permission from ref 33. Copyright 2015. John Wiley and Sons.

### 2.3 Fabrication of the All-Solid-State F-SC Device

Following the fabrication of the electrodes, the F-SC device was assembled by using a gel electrolyte composed of PVA-H_3_PO_4_. The gel electrolyte was fabricated by mixing PVA (6 g) in deionized (DI) water (60 mL) under constant stirring at 85 °C until a transparent solution was formed. Subsequently, 1 M H_3_PO_4_ was gradually introduced into the transparent PVA solution in a 1:1 ratio, followed by continuous stirring for an additional 30 min to ensure homogeneous mixing. To facilitate proper absorption of the electrolyte, the ZMO@carbon yarn electrodes were immersed in the prepared gel electrolyte solution for 1 h. The electrodes were then removed and dried at room temperature to facilitate the formation of a stable gel layer over them, as displayed in Figure 1d. After the initial drying, two gel-coated ZMO@carbon yarn electrodes were twisted to form a fiber-shaped device structure. To ensure optimal ionic conductivity and mechanical integrity, an additional layer of gel electrolyte was coated over the twisted electrodes, followed by another drying period of 10 h at room temperature. This step ensures that any remaining gaps between the electrodes are adequately filled with the electrolyte, as depicted in Figure 1e. During the assembly process, multiple layers of the gel electrolyte were carefully coated to maintain a uniform thickness across the device. However, as this is a manual fabrication process, slight variations in electrolyte thickness and fiber circumference may occur, although they remain within a controllable range. The gel electrolyte serves a dual purpose as both an ion-conducting medium and a separator, preventing direct contact between the ZMO@carbon yarn electrodes while facilitating efficient charge transfer. The final assembled F-SC device had a total length of approximately 6 cm after the electrodes were twisted together. The fabricated F-SC device can be knitted in wearable fabrics and utilized in wearable textile electronics applications, as presented in Figure 1f,g.

### 2.4 Characterization

The surface morphology of fabricated ZMO@carbon yarn electrodes was examined by using a field-emission scanning electron microscope (FE-SEM, MIRA-3 from Tescan), which provides high-resolution imaging to analyze the structural integrity, uniformity, and distribution of the deposited ZnMn_2_O_4_ nanostructures. The crystallinity and phase composition of the fabricated electrodes were determined by using X-ray diffraction (XRD) analysis with a Rigaku X-ray diffractometer (Cu Kα radiation source). XRD characterization ensures that the desired crystalline phase is achieved without unwanted secondary phases. A two-electrode configuration was used to evaluate the electrochemical performance of the fabricated F-SC device. Elemental composition analysis was conducted using energy-dispersive X-ray spectroscopy (EDS, Thermo Fisher Apreo S LoVac). The specific surface area of the sample was determined using a Brunauer–Emmett–Teller (BET) surface area analyzer (ASIQWIN Quantachrome) operating at 77 K. Raman scattering spectra for the ZMO@carbon yarn electrode were recorded at room temperature using a RENISHAW Raman spectrometer with a 514 nm Ar-ion laser, spanning a wavenumber range of 100–2000 cm^–1^. The oxidation states of the elements present were analyzed by X-ray photoelectron spectroscopy (XPS) by using a PHI 5000 Versa Probe III instrument. The CV and galvanostatic charge/discharge (GCD) measurements were conducted using a Metrohm Autolab Potentiostat/Galvanostat (MAC-80,039) instrument to analyze the charge storage behavior, specific capacitance, rate capability, and energy density of the F-SC device. CV measurements were conducted over a scan rate range of 10–100 mV/s, while GCD measurements were carried out at current densities ranging from 0.1 to 0.5 mA/cm^2^ within a voltage window of 0–1.5 V.

To assess the long-term durability of the F-SC devices, the device was subjected to 10,000 consecutive charge–discharge cycles at a current density of 0.5 mA/cm^2^. The bending stability of the F-SC device was also evaluated by performing CV measurements at a scan rate of 10 mV/s before and after extreme twisting deformation to measure the mechanical robustness and reliability of the device under real-world conditions. Electrochemical impedance spectroscopy (EIS) was employed to measure the internal resistance and charge transport properties of the fabricated F-SC device. To assess charge-transfer resistance and ion diffusion properties, impedance measurement was performed by applying a 5 mV AC voltage across a frequency range from 100 kHz to 0.01 Hz. Standard electrochemical equations are used to calculate the electrochemical performance metrics of the F-SC device, such as the areal capacitance (*C*_A_), areal energy density (*E*_A_), and areal power density (*P*_A_). The areal capacitances are derived from both CV and GCD data using eqs 1 and 2, respectively, while the energy and power densities of the device were calculated using eqs 3 and 4, respectively. These parameters offer a detailed understanding of the energy storage capabilities and practical applicability of the fabricated ZMO@carbon-yarn-based F-SC devices

1 $$C_{A}\;\left(F/{\mathrm{cm}}^{2}\right)=\frac{\int_{-V}^{V}idV}{V\left(\frac{dV}{dt}\right)A_{\mathrm{device}}}$$

2 $$C_{A}\;\left(F/{\mathrm{cm}}^{2}\right)=\frac{I\Delta t}{V}$$

3 $$E_{A}\;\left(\mathrm{Wh}/{\mathrm{cm}}^{2}\right)=\frac{1}{2\times 3600}C_{A}V^{2}$$

4 $$P_{A}\;\left(W/{\mathrm{cm}}^{2}\right)=\frac{E_{A}}{\Delta t}\times 3600$$

## 3 Results and Discussion

The FE-SEM image of the bare carbon yarn before electrodeposition is displayed in Figure 2a, which shows that the surface of the bare carbon yarn appears smooth and homogeneous compared to the carbon yarn after the growth of ZnMn_2_O_4_. The morphological characteristics of the fabricated ZMO@carbon yarn electrodes are displayed in Figure 2b–d. Following the electrodeposition process, a uniform growth of ZnMn_2_O_4_ nanostructures can be observed on the surface of individual carbon yarn, as depicted in Figure 2b. The deposited ZnMn_2_O_4_ forms a well-adhered, homogeneous coating with an approximate diameter of 10 μm for a singular yarn. Moreover, the high-magnification FE-SEM images of the deposited ZMO@carbon yarn electrodes (Figure 2c,d) reveal the interconnected nanoarchitecture of ternary ZnMn_2_O_4_ on the carbon yarn surface. The interconnected network structure may promote rapid ionic diffusion and improve the mechanical integrity of the composite electrode, which are crucial for long-term cycling stability and flexibility in energy storage devices. Additionally, the elemental mapping of the ZMO@carbon yarn electrode is performed using EDS, as shown in Figure 2e, which confirms the uniform distribution of Zn, Mn, and O elements on the surface of the carbon yarn. The crystalline structure and information about the phase of the synthesized ZnMn_2_O_4_ nanostructures were assessed through the XRD technique. All diffraction peaks of the fabricated ZMO@carbon yarn electrode are well-matched with the tetragonal phase ZnMn_2_O_4_ standard pattern published in the literature (JCPDS card No. 24-1133), as displayed in the XRD pattern in Figure 2f.^34,35^ The deposited ZnMn_2_O_4_ nanostructures exhibit high purity, as evidenced by the absence of peaks corresponding to secondary phases, which are consistent with previously reported ZnMn_2_O_4_ nanomaterials in energy storage applications.^34,35^ Additionally, diffraction peaks (star marked) at 2θ values of 25.8 and 43°, corresponding to the (002) and (100) planes, respectively, are attributed to the carbon yarn substrate,^36,37^ indicating that the deposition process maintains the structural integrity of the underlying carbon framework. The XRD pattern of the ZMO@carbon yarn electrodes was fitted and refined by Rietveld refinement using FullProf software, as displayed in Figure 2g, and Table 1 shows the corresponding parameters. The measured lattice parameters of ZnMn_2_O_4_ (*a* = *b* = 5.72200 Å, *c* = 9.29066 Å) and the carbon yarn substrate (*a* = *b* = 2.50783 Å, *c* = 6.95675 Å) exhibit strong agreement with the refined fitting parameters, including the goodness of fitting (χ^2^) = 1.86, *R*-profile factor (*R*_p_, %) = 6.90, and *R*-weighted pattern (*R*_wp_, %) = 7.13. Further, the crystal structure of ZnMn_2_O_4_ generated from the refined data is displayed in Figure 2h. The crystal structure depicts Zn, Mn, and O atoms as gray, violet, and red spheres, respectively, where Zn atoms are situated in tetrahedral sites, while Mn atoms occupy octahedral sites. Furthermore, an XPS analysis was performed to investigate the elemental composition and chemical states of the fabricated ZMO@carbon yarn electrode. The XPS survey spectra of the electrode, presented in Figure S1, confirm the presence of Zn, Mn, O, and C, further verifying the purity of the fabricated electrode.

**Figure 2:**
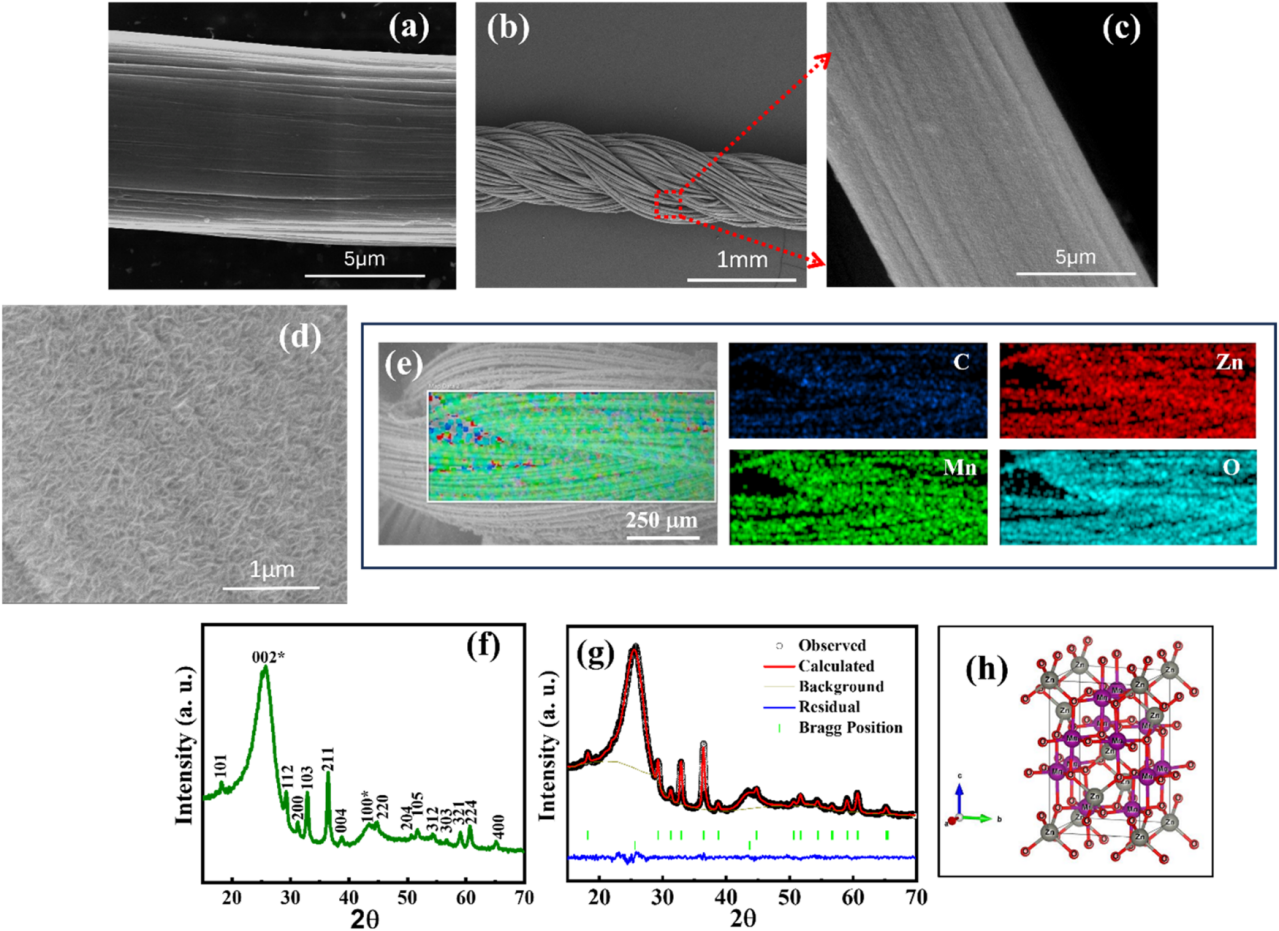
(a) FE-SEM image of bare carbon yarn, (b) FE-SEM image of the ZnMn_2_O_4_-grown carbon yarn electrode via electrodeposition, (c, d) high-magnification images and (e) elemental mapping of the surface of ZnMn_2_O_4_-grown carbon yarn electrode, (f) XRD spectra of ZnMn_2_O_4_-grown carbon yarn electrode, (g) Rietveld refined XRD pattern, and (h) crystal structure of ZnMn_2_O_4_ nanostructures.

**Table 1:** Refined Crystallographic Parameters of the ZMO@carbon Yarn Electrode

elements	*x*	*y*	*z*	occupancy	temperature factor
Zn	0	0	0	0.124	1.46
Mn	0	0.25	0.625	0.264	2.06
O	0	0.23106	0.38391	0.521	2.445
C1	0	0	0	0.151	1.334
C2	0.33330	0.66670	0.00500	0.149	1.484

Moreover, the Raman spectra of the ZMO@carbon yarn electrode exhibit distinct peaks between 300 and 700 cm^–1^, corresponding to ZnMn_2_O_4_, as shown in Figure S2­(a). Overall, the presence of these peaks confirms the successful synthesis of ZMO@carbon yarn and the coexistence of Zn, Mn, O, and C elements in the electrode.^38^ Further, nitrogen adsorption–desorption measurements of the ZMO@carbon yarn electrode at 77 K, depicted in Figure S2­(b), reveal that the sample exhibits a type-IV isotherm over the relative pressure (*P*/*P*_0_) range of 0.0–1.0, characteristic of a mesoporous structure of ZnMn_2_O_4_.^39^

Electrochemical analysis of the flexible F-SC device is systematically performed in a two-electrode configuration to evaluate its charge storage capabilities. CV measurements are conducted at different scan rates from 10 to 100 mV/s within a potential window of 0–1.5 V, as demonstrated in Figure 3a. The quasi-rectangular shape of the CV curves across all scan rates signifies the typical pseudocapacitive charge storage behavior, which is attributed to the fast and reversible redox reactions occurring within the ZnMn_2_O_4_ nanostructures, facilitating efficient charge storage.^37^ Further, GCD measurements are conducted at a range of current densities from 0.1 to 0.5 mA/cm^2^, as represented in Figure 3b. The GCD curves display a nearly triangular and symmetric profile across all current densities, supporting the CV results and further confirming the pseudocapacitive charge storage mechanism.^40^ The linear voltage–time relationship suggests minimal internal resistance and high reversibility of the redox reactions, which are essential for achieving stable energy storage performance. Such behavior is consistent with prior reports on ZnMn_2_O_4_-based SCs, where the redox-active nature of the active material plays a crucial role in efficient charge storage.^41,42^

**Figure 3:**
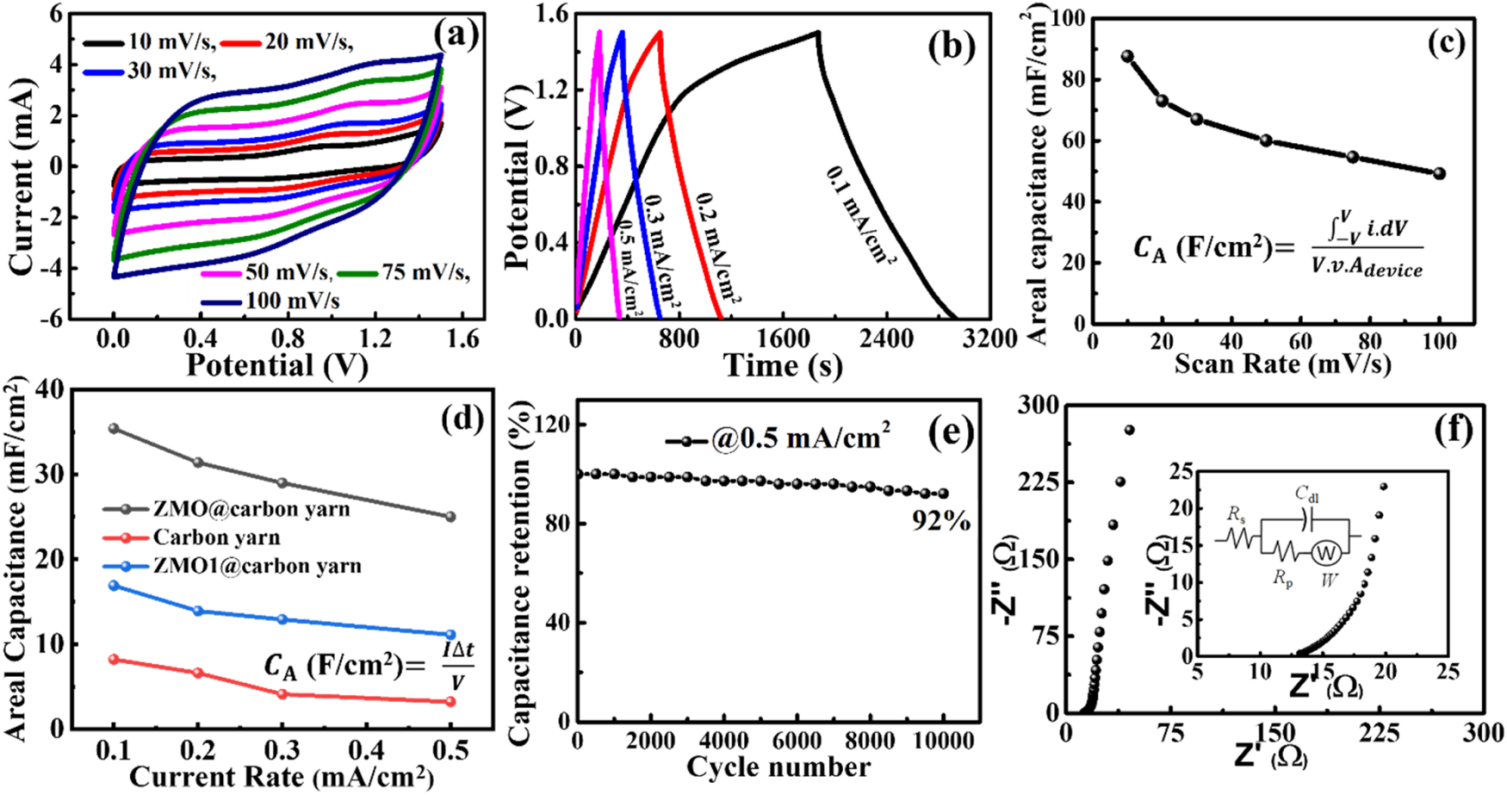
Electrochemical analysis of the F-SC device, (a) CV analysis at the scan rates of 10–100 mV/s, (b) GCD analysis at current rates of 0.1–0.5 mA/cm^2^, (c) areal capacitance vs. scan rate, (d) areal capacitance vs current rate, (e) long cyclability analysis of the F-SC device, and (f) EIS analysis of the F-SC device; inset displays the magnified EIS spectra and the electrical equivalent circuit.

To assess the rate capability of the flexible F-SC device, the areal capacitances are evaluated at various scan rates and current densities using eqs 1 and 2, as displayed in Figure 3c and d, respectively. The areal capacitance vs. scan rate graph displayed the maximum areal capacitance of 87.6 mF/cm^2^ at a scan rate of 10 mV/s in Figure 3c. Additionally, the device delivered areal capacitances of 73, 67, 60, 54.6, and 49.2 mF/cm^2^ at scan rates of 20, 30, 50, 75, and 100 mV/s, respectively. As the scan rate increases, gradually reduced capacitance values are observed because, at higher scan rates, electrolyte ions do not have enough time to diffuse to the internal pores of the electrode, thus, limiting the redox reaction. The electrochemical reactions are unable to keep up with the rapid potential changes, leading to incomplete utilization of all available active sites.^42^ Similarly, Figure 3d presents the areal capacitance versus current density graph, which exhibits maximum capacitance values of 35.4, 31.4, 29, and 25 mF/cm^2^ at current densities of 0.1, 0.2, 0.3, and 0.5 mA/cm^2^, respectively. Further, the device fabricated using ZMO1@carbon yarn electrodes offered a maximum areal capacitance of 16.9 mF/cm^2^ at current densities of 0.1 mA/cm^2^. A reduction in capacitance for the device fabricated based on ZMO@carbon yarn at higher values of current rates is primarily attributed to the insufficient penetration of electrolytic ions into the bulk of the active material, which limits the availability of electrochemically active redox sites. This trend is consistent with previous studies on metal-oxide-based SCs, where the rate-dependent capacitance reduction is attributed to the kinetic limitations of ion transport.^40^

Cyclic stability is a critical factor in determining the long-term performance and practical applicability of energy storage systems, which are expressed through capacity retention (%). The cycling stability of the F-SC device is evaluated through GCD measurements at a current density of 0.5 mA/cm^2^ for 10,000 cycles, as displayed in Figure 3e. After 10,000 cycles, the device retained 92% of its original capacitance, showcasing good electrochemical stability and structural durability. The lesser capacitance decay indicates that the ZnMn_2_O_4_ nanostructures remain electrochemically active and stable throughout continuous charge–discharge cycles. Further, the good capacitance retention is attributed to the mechanical integrity of the ZMO@carbon yarn electrode fabricated via electrodeposition.

To further investigate the charge transport dynamics and interfacial properties of the fabricated flexible F-SC device, EIS analysis is conducted over a frequency range of 100 kHz to 0.01 Hz with an applied AC voltage of 5 mV, as illustrated in Figure 3f. The Nyquist plot obtained from EIS measurements is fitted using ZView software based on an appropriate electrical equivalent circuit,^43^ as illustrated in the inset of Figure 3f. In the equivalent circuit model, the solution resistance (*R*_s_) represents the inherent ohmic resistance of the electrolyte and electrode material, while the charge-transfer resistance (*R*_p_) denotes the faradaic charge-transfer resistance at the interface of the electrode–electrolyte, which are critical factors influencing the redox reaction kinetics.^44,45^ The double-layer capacitance (*C*_dl_) accounts for the capacitance generated by the electrical double layer that forms at the interface between the electrode and the electrolyte. Additionally, the Warburg impedance (*W*) indicates ion diffusion resistance within the porous ZnMn_2_O_4_ nanostructures and provides insight into the mass transport limitations of the system. By fitting the EIS data, the extracted values for *R*_s_, *R*_p_, *C*_dl_, and *W* are found to be 13.9, 0.02 Ω/cm^2^, 4.44 × 10^–5^ F/cm^2^, and 5.2 Ω/cm^2^, respectively. The equivalent series resistance (ESR) of the device, resulting from the combined contribution of *R*_s_ and *R*_p_, is calculated to be 13.92 Ω/cm^2^. This low ESR value within flexible solid-state devices signifies excellent charge transport properties and reduced internal resistance, which are critical for achieving high power density and rapid charge–discharge performance.^46^ The electrochemical performance of the device is attributed to the dense and uniform growth of ZnMn_2_O_4_ nanostructures on the carbon yarn, forming a highly conductive and electrochemically active interface and the synergic contribution of both carbon and metal oxide. The interconnected morphology of the ZnMn_2_O_4_ network minimizes energy losses and enhances the effective contact area between the electrode and electrolyte, thereby facilitating rapid ion transport and charge storage. The fabricated flexible F-SC device exhibits a slightly higher ESR compared to conventional SCs utilizing liquid electrolytes. This increase in ESR is primarily attributed to the use of a solid-state electrolyte (PVA-H_3_PO_4_), which, while offering structural advantages, typically has a lower ionic conductivity than its liquid counterparts. Similar trends have been observed in previous studies on solid-state SCs, where the trade-off between ionic transport efficiency and mechanical stability is a crucial design consideration for practical applications.^47^ The utilization of the PVA-H_3_PO_4_-based solid-state electrolyte offers several application-oriented advantages, particularly for wearable and flexible electronics.^48^ Notably, it eliminates the risk of electrolyte leakage, ensuring long-term stability and reliability, and enhances interfacial adhesion between the electrode and electrolyte, which, in turn, improves the mechanical strength of the device. This strong adhesion contributes to the device’s ability to withstand bending and twisting, making it well-suited for applications in flexible and wearable electronics.

To gain further information on the charge storage mechanism of the fabricated flexible F-SC device, the Dunn method^43^ is employed to distinguish the contributions of diffusion-controlled redox reactions and capacitive surface reactions to the overall capacitive response of the device. This method is crucial for understanding the relative dominance of Faradaic (pseudocapacitive) and non-Faradaic processes (electric double layer (EDL)) in the mechanism for energy storage. As displayed in Figure 4a, the nearly linear relationship observed in the current (*i*) vs square root of the scan rate (ν^1/2^) plot validates the presence of surface-dominated redox reactions, thereby validating the pseudocapacitive charge storage behavior of the ternary ZnMn_2_O_4_ nanostructures. Additionally, the *y*-axis intercept in the *i* versus ν^1/2^ plot represents the capacitive current contribution, primarily arising from the double-layer capacitance at the interface of electrode–electrolyte, which is attributed to the carbon yarn substrate.^49^ Furthermore, a quantitative assessment of the diffusion-controlled and capacitive contributions to the total stored charge is conducted at a 50 mV/s scan rate, as illustrated in Figure 4b. The blue-shaded region represents the diffusion-controlled current contribution, whereas the brown-shaded region corresponds to the capacitive current contribution. The capacitive and diffusion-based charge storage mechanisms are further analyzed across a range of scan rates between 10 and 100 mV/s, as is evident in Figure 4c. A clear trend is observed where the capacitive contribution increases with higher scan rates, indicating a shift toward a predominantly surface-controlled charge storage mechanism at higher scan rates. This phenomenon suggests that the ZnMn_2_O_4_ nanostructures grown on carbon yarns provide rapid charge-transfer kinetics and a superior rate capability, which are essential for high-performance SC applications.

**Figure 4:**
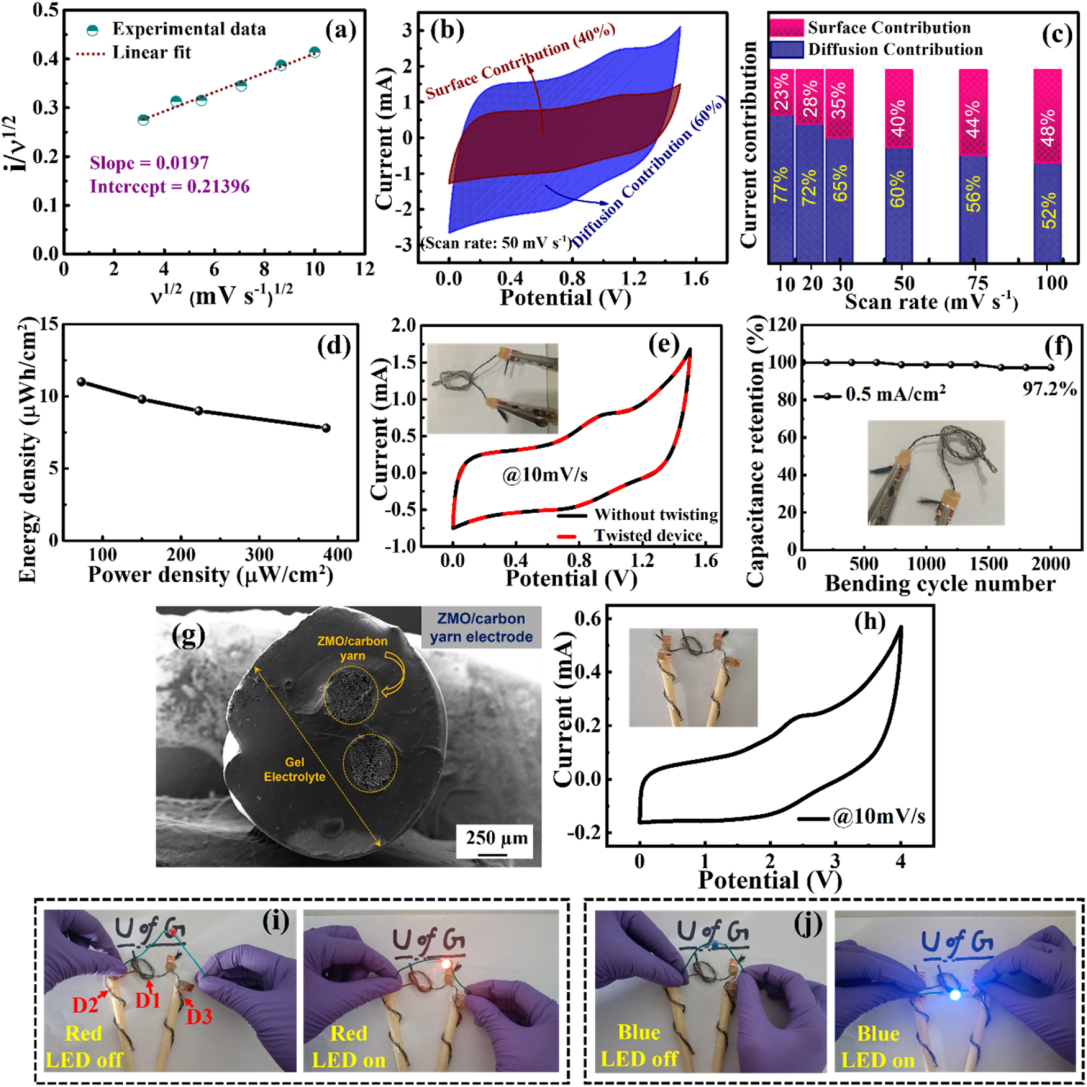
(a) *i* vs ν^1/2^ plot for the F-SC device, (b) capacitive and diffusive current contribution at 50 mV/s scan rate, (c) bar chart with diffusion and capacitive current contributions (in %) at scan rates between 10 and 100 mV/s, (d) Ragone plot displaying the energy density vs power density for the F-SC device, (e) CV analysis of the F-SC device before and after twisting at 10 mV/s scan rate, (f) long-term cyclability analysis of the F-SC device over 2000 bending cycles, (g) cross-sectional FE-SEM of the flexible F-SC device after 10,000 cycles, (h) CV analysis of three series-connected F-SCs at 10 mV/s scan rate, and (i, j) three series-connected F-SC devices glowing a red and blue LED, respectively, during extreme bending and twisting conditions.

The most essential metrics for evaluating the performance and practical viability of SC devices are the energy and power densities. These metrics determine the capability of an SC device to deliver continuous energy while maintaining a high power output, making them essential for practical applications, particularly in wearable and flexible electronics. Energy density (*E*) and power density (*P*) for the flexible F-SC device are calculated using eqs 3 and 4, respectively, and are graphically represented in the Ragone plot in Figure 4d. A single flexible F-SC device delivers an impressive energy density ranging from 7.8 to 11 μWh/cm^2^ and a power density between 74 and 385 μW/cm^2^. These performance metrics surpass those of many existing flexible fiber and yarn-based SCs reported in the literature, particularly those utilizing metal oxide/sulfide materials (especially manganese oxides), tabulated in Table 2.^10,50–53^ The good energy and power performance of the F-SC device are ascribed to the high conductivity of the carbon yarn substrate, coupled with the efficient charge storage properties of the ZnMn_2_O_4_ nanostructures grown over it. The synergy between the double-layer capacitance of the carbon yarns and the pseudocapacitive behavior of ZnMn_2_O_4_ ensures an optimal balance between the energy storage capability and fast charge–discharge kinetics.

**Table 2:** Performance Comparison of Flexible F-SC Devices Reported in the Literature

active material	substrate	preparation method	capacitance	energy density (μWh/cm^2^)	power density	refs
MnCo_2_O_4_	Cu wire	electrodeposition	20.6 mF/cm (54.8 mF/cm^2^)	12.8	110 μW/cm^2^	^10^
reduced graphene oxide (rGO)	cotton yarns	hydrothermal	2.99 mF/cm (9.54 mF/cm^2^) at 0.02 mA/cm	1.32	31.84 μW/cm^2^	^50^
MnO_2_ and MoO_3_	carbon fiber	electrodeposition	4.86 mF/cm^2^ at 0.5 mA/cm^2^	2.70	0.53 mW/cm^2^	^51^
MoS_2_/MnS	graphene nanoribbon	laser-induced process	58.3 mF/cm^2^ at 50 μA/cm^2^	7.0	49.9 μW/cm^2^	^52^
Cu-MOF	rGO fiber	wet-spinning and HI chemical reduction	44.6 mF/cm^2^ at 5 mV/s	0.51	2.54 μW/cm^2^	^53^
ZnMn_2_O_4_	carbon yarn	electrodeposition	87.6 mF/cm^2^ at 10 mV/s and 35.4 mF/cm^2^ at 0.1 mA/cm^2^	11	385 μW/cm^2^	this work

To serve effectively as a power source for wearable electronic systems, the flexible F-SC device must exhibit excellent mechanical stability, enabling direct integration into garments through knitting or weaving processes; maintaining stable electrochemical performance under mechanical stress is a crucial requirement for next-generation flexible energy storage systems. To evaluate the bending stability of the device, CV measurement is performed at a scan rate of 10 mV/s before and after mechanical deformation, such as twisting, as presented in Figure 4e. The nearly identical CV curves recorded before and after twisting demonstrate that the device retains its capacitive performance despite undergoing mechanical stress. Additionally, the bending cyclability analysis of the device is performed for 2000 bending cycles at 0.5 mA/cm^2^, displayed in Figure 4f. The device retained 97.2% capacitance retention after 2000 cycles under bending conditions. Further, the postcycling cross-sectional FE-SEM characterization for the flexible F-SC device has been performed after 10,000 cycles, displayed in Figure 4g. The FE-SEM image indicates that the device retains its mechanical structure, consisting of a gel electrolyte around the ZMO@carbon yarn electrodes. These observations suggest that the strong adhesion between the ZnMn_2_O_4_ nanostructures and carbon yarns, combined with the flexibility of the solid-state electrolyte, allows the device to withstand bending and twisting without compromising its electrochemical functionality.

To further evaluate the feasibility of the flexible F-SC device as a power source for real-world textile applications, its ability to power electronic components is examined in flexible and wearable applications. Three identical F-SC devices are connected in series and subjected to various mechanical deformations, such as bending and twisting. To assess the total voltage window of the series-connected F-SC devices, CV measurements are performed at a 10 mV/s scan rate within a voltage range between 0 and 4 V, as illustrated in Figure 4h. Three series-connected F-SC devices display a quasi-rectangular CV curve between 0 and 4 V, representing the pseudocapacitive charge storage mechanism and the stable electrochemical response of the interconnected SC devices. The ability of the series-connected devices to maintain a stable capacitive profile under bending and twisting reinforces their mechanical durability and reliability for flexible energy storage applications. The three series-connected F-SC devices are used to further assess the practical energy output to power a red LED under different mechanical deformation conditions, as shown in Figure 4i. The devices successfully illuminated the red LED for over 5 min across different mechanical deformations, demonstrating consistent power delivery. Additionally, the same setup was able to power a blue LED for 2 min under similar conditions (Figure 4j), further validating the high energy retention and stable performance of the fabricated SC devices. The Supporting Video demonstration (S1) also confirms the device’s capability to function reliably in dynamic and wearable environments. The outcomes of this investigation demonstrate the significant promise of the flexible F-SC device for integration into next-generation smart textiles and wearable electronics. The combination of high energy and power density, mechanical durability, and long-term electrochemical stability under deformation meets the crucial requirements of flexible and low-power electronic applications. These results contribute to ongoing progress and open up new possibilities for further evolution of fiber-based energy storage systems, enabling seamless integration into smart textiles for applications in next-generation self-powered wearable electronics.

## 4 Conclusions

This study successfully fabricates a highly flexible and twistable F-SC device using a one-step electrodeposition process to grow ternary metaloxide nanostructures (ZnMn_2_O_4_) directly onto conductive carbon yarn substrates. The uniform growth of ZnMn_2_O_4_ nanostructures on a conductive carbon yarn substrate effectively enhances the capacitive performance of the fabricated device while ensuring good mechanical integrity, allowing for superior bending and structural stability. The fabricated device demonstrated excellent electrochemical performance, exhibiting an areal capacitance of 87.6 mF/cm^2^ at a scan rate of 10 mV/s and 35.4 mF/cm^2^ at a current density of 0.1 mA/cm^2^. Further analysis using the Dunn method at a moderate scan rate (50 mV/s) revealed that the areal capacitance is composed of 40% EDL capacitance and 60% pseudocapacitance, demonstrating a synergistic effect between the ZnMn_2_O_4_ nanostructures and the carbon yarn substrate. The device also exhibited excellent stability and durability, maintaining 92% of its original capacitance after 10,000 charge–discharge cycles with no significant performance degradation. Moreover, the device achieved an energy density of 11 μWh/cm^2^ and a power density of 385 μW/cm^2^, maintaining consistent performance under extreme bending and twisting conditions. The superior electrochemical stability and mechanical flexibility of the device are attributed to the uniform and robust growth of ZnMn_2_O_4_ nanostructures, which promote efficient charge storage through a pseudocapacitive mechanism while maintaining strong adhesion to the carbon yarns. By integrating high-performance ternary materials with mechanically resilient and twistable electrode architectures using a streamlined electrodeposition method, this work showcases a promising strategy for designing flexible and durable SCs that are ideally suited for seamless integration into next-generation wearable and portable electronic devices.

## Supplementary Material





## Data Availability

Data will be made available on request.

## Notes

Device’s capability to function reliably in dynamic and wearable environments (Video S1) (MP4)
XPS analysis of the ZMO@carbon yarn electrode, Raman spectra of the ZMO@carbon yarn electrode, nitrogen adsorption–desorption measurements of the ZMO@carbon yarn electrode, and pore size distribution of the ZMO@carbon yarn electrode (PDF)
