# Supplementary material for: Flexible and Twistable ZnMn2O4‑Electrodeposited Yarn Supercapacitors for Wearable Electronics
Source: ACS Appl Mater Interfaces. 2025 Jun 25;17(27):39108–17. doi: 10.1021/acsami.5c06545 (PMC12257461; doi:10.1021/acsami.5c06545)
Supplement: Supplementary file 2 [file am5c06545_si_002.pdf]

Supporting Information

Flexible and twistable $ZnMn_2O_4$-electrodeposited yarn supercapacitors for wearable electronics

Shalu Rani[1, 2*], Gaurav Khandelwal[2], Abhinav Tandon[3], Sanjay Kumar[1], Akshaya Kumar Aliyana[4], Suresh C. Pillai[5], George K. Stylios[4], Nikolaj Gadegaard[2], Daniel M. Mulvihill[2*]

[1]Department of Electronics Engineering, Indian Institute of Technology (Indian School of Mines), Dhanbad, Jharkhand, 826004, India
[2]James Watt School of Engineering, University of Glasgow, Glasgow, G12 8QQ, UK
[3]Department of Mechanical Engineering, College of Design and Engineering, National University of Singapore, Singapore, 117575, Singapore
[4]Research Institute for Flexible Materials, Heriot-Watt University (Scottish Borders Campus), Galashiels, TD1 3HF, UK
[5]Nanotechnology and Bio-Engineering Research Group, Department of Environmental Science, Atlantic Technological University, Sligo, F91 YW50, Ireland

*Corresponding authors: Shalu@iitism.ac.in, Daniel.Mulvihill@glasgow.ac.uk.

The XPS analysis has been performed to investigate the elemental composition and chemical states of the ZMO@carbon yarn electrode. The survey spectra (see Fig. S1 (a)) of the as-fabricated electrode confirm the presence of Zn, Mn, O, and C elements. The high-resolution Zn2p XPS spectra for the ZMO@carbon yarn electrode (see Fig. S1 (b)) exhibit two prominent peaks at binding energies of 1020.90 eV and 1043.97 eV, corresponding to $Zn2p_{3/2}$ and $Zn2p_{1/2}$, respectively.[1] Likewise, the Mn2p spectra (see Fig. S1 (c)) reveal two different oxidation states of Mn, indicated by $Mn2p_{3/2}$ (641.26 eV) and $Mn2p_{1/2}$ (653.06 eV), with a spin-orbit splitting of approximately 11.8 eV. Gaussian deconvolution identifies these peaks as Mn^{3+} (641.09 and 652.74 eV) and Mn^{2+} (642.63 and 654.76 eV), respectively.[2] The O1s XPS spectra (see Fig. S1 (d)) display three principal peaks at 529.68, 531.68, and 532.75 eV, corresponding to lattice oxygen (O_L), oxygen-deficient sites (O_d), and surface-adsorbed water (O_W), respectively.[3] Additionally, the C 1s XPS signals (see Fig. S1 (e)) from the ZMO@carbon yarn sample confirm the presence of a carbonaceous support. Distinct peaks near 284.71 eV and 288.04 eV can be attributed to C=C and O–C=O bonds, respectively.[4, 5] These findings confirm that the carbon matrix is well-integrated in the yarn electrode, providing conductive pathways that enhance the electrode's electrochemical performance. Collectively, these XPS results support

the successful formation of a ZMO@carbon yarn electrode with the desired oxidation states of Zn and Mn, as well as the presence of lattice oxygen, oxygen vacancies, and a supportive carbon matrix.

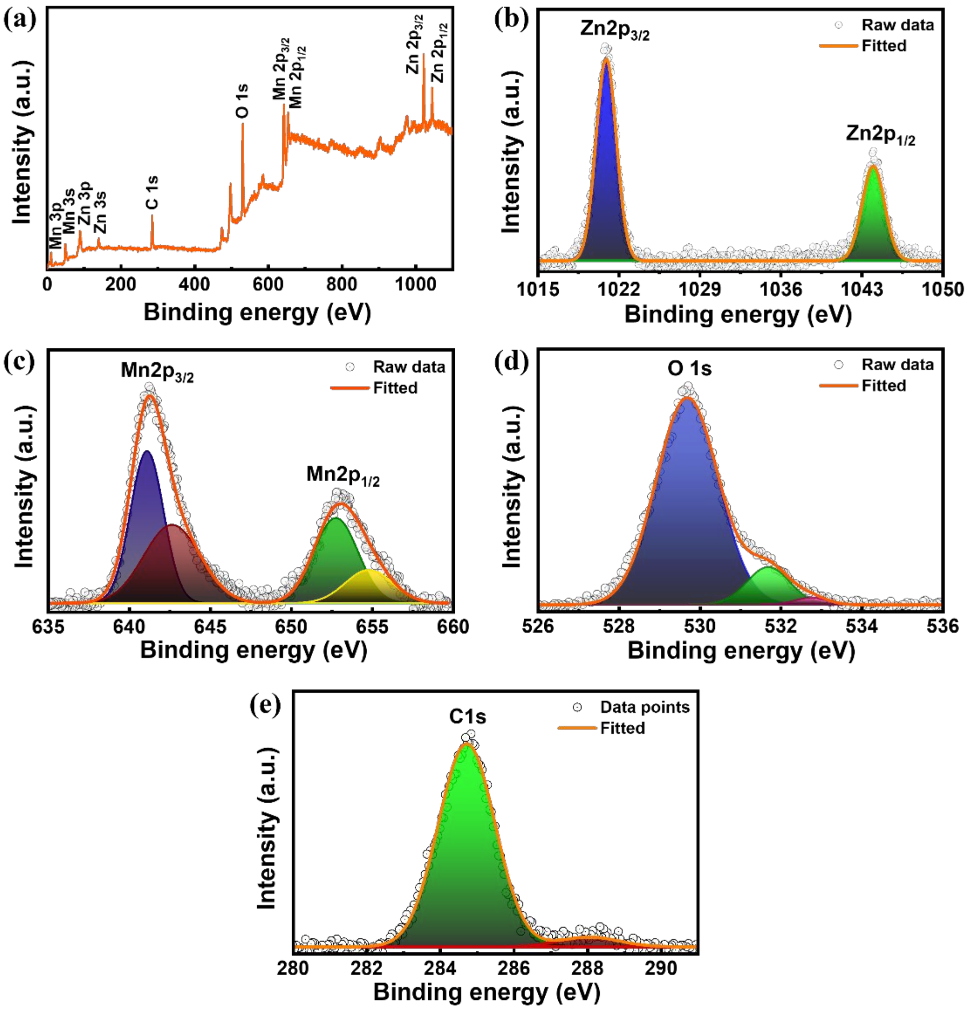

Fig. S1: (a) XPS survey spectrum, and high-resolution XPS spectra of (b) Zn2p, (c) Mn2p, (d) O1s, and (e) C1s, of ZMO@carbon yarn electrode.

The Raman spectra of the ZMO@carbon yarn electrode, presented in Fig. S2 (a), exhibit distinct peaks between 300 and 700 cm^{-1} corresponding to the $ZnMn_2O_4$. Specifically, the peaks at around 324 and 385 cm^{-1} are associated with the vibrational modes of MnO_6 octahedral sites, indicative of Mn–O stretching, while the peak at 680 cm^{-1} corresponds to the A_{1g} symmetric breathing mode of ZnO_4 tetrahedra, reflecting Zn–O stretching in the tetrahedral site.[1, 6] Additionally, broad peaks at about 1348 and 1583 cm^{-1} represent the defect-induced (D) mode and graphitization-induced (G) mode of amorphous carbon, respectively. The

intensity ratio of the D-to-G bands serves as an indicator of the graphitization degree in carbon-based materials; the observed I_D/I_G ratio of approximately 0.851 suggests relatively low graphitization and a higher degree of defects in the electrode. Overall, the presence of these peaks confirms the successful synthesis of ZMO@carbon yarn and the coexistence of Zn, Mn, O, and C elements in the electrode.[1]

Furthermore, the Nitrogen adsorption-desorption measurements of the ZMO@carbon yarn electrode at 77 K, depicted in Fig. S2 (b), reveal that the sample exhibits a type-IV isotherm over the relative pressure (P/P_0) range of 0.0–1.0, characteristic of a mesoporous structure of $ZnMn_2O_4$.[7, 8] The density functional theory (DFT) pore size distribution (PSD) shown in Fig. S2 (c) pinpoints a dominant pore size of about 2.897 nm, confirming mesoporosity within the sample. The BET analysis further indicates a specific surface area of 30.24 $m^2\ g^{-1}$ and a total pore volume of 0.084 $cm^3\ g^{-1}$ at P/P_0 = 1.0. The mesoporosity, alongside the elevated surface area, conceivably increases the number of accessible reactive sites, thereby enhancing adsorption processes at the electrode-electrolyte boundary.[7, 8]

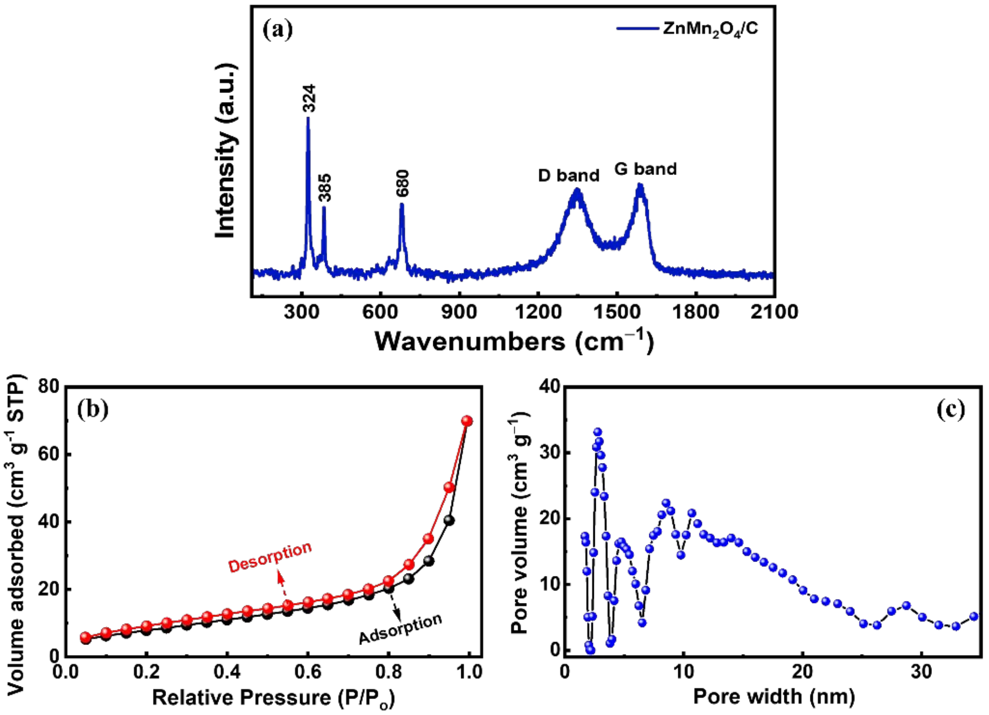

Fig. S2: (a) Raman spectra, (b) multipoint nitrogen adsorption-desorption isotherm, and (c) pore size distribution of ZMO@carbon yarn electrode.

References:

(1) Tang, Q.; Shi, Y.; Ding, Z.; Wu, T.; Wu, J.; Mattick, V.; Yuan, Q.; Yu, H.; Huang, K. Three-dimensional hierarchical graphene and CNT-coated spinel $ZnMn_2O_4$ as a high-stability anode for lithium-ion batteries. *Electrochimica Acta* **2020**, *338*, 135853.

(2) Tandon, A.; Sharma, Y. In Situ Electrophoretic Decorated Cactus-Type Metallic-Phase MoS_2 on $CaMn_2O_4$ Nanofibers for Binder-Free Next-Generation LIBs. *Applied Materials & Interfaces* **2024**, *16* (14), 17728-17744.

(3) Samage, A.; Kuppe, P.; Halakarni, M.; Ganesan, B. K.; Kamath, S. V.; Yoon, H.; Kotrappanavar, N. S. Room temperature and rapid synthesis of $ZnMn_2O_4$ nanostructured spinel using deep eutectic solvent for high energy asymmetric supercapacitors. *Journal of Energy Storage* **2024**, *97*, 112934.

(4) Biesinger, M. C. Applied Surface Science Accessing the robustness of adventitious carbon for charge referencing (correction) purposes in XPS analysis: Insights from a multi-user facility data review. *Applied Surface Science* **2022**, *597*, 153681.

(5) Chen, J.; Zhuang, Y.; Qiao, Y.; Zhang, Y.; Yuan, A.; Zhou, H.; Co/Co_7Fe_3 heterostructures with controllable alloying degree on carbon spheres as bifunctional electrocatalyst for rechargeable zinc–air batteries. *Int. J. Miner. Metall. Mater.*, **2025**, *32*, 476-487.

(6) Zhang, T.; Yue, H.; Qiu, H.; Zhu, K.; Zhang, L.; Wei, Y.; Du, F.; Chen, G.; Zhang, D. Synthesis of graphene-wrapped $ZnMn_2O_4$ hollow microspheres as high performance anode materials for lithium ion batteries. *RSC Advances* **2015**, *5*, 99107–99114.

(7) Chen, J.; Zuo, H.; Wang, C.; Zhang, Y.; Gao, W.; Zhao, N.; Huang, Y.; Xiao, S. Synthesis and electrochemical properties of $ZnMn_2O_4$ with hollow porous panpipe-like structure as anode material for Li-ion battery. *Electrochimica Acta* **2022**, *426*, 140780.

(8) Zhao, L.; Li, X.; Zhao, J. Fabrication, characterization and photocatalytic activity of cubic-like $ZnMn_2O_4$. *Applied Surface Science* **2013**, *268*, 274–277.
